# Dendritic spine and synapse pathology in chromatin modifier-associated autism spectrum disorders and intellectual disability

**DOI:** 10.3389/fnmol.2022.1048713

**Published:** 2023-01-19

**Authors:** Thomas James L. Ford, Byeong Tak Jeon, Hyunkyoung Lee, Woo-Yang Kim

**Affiliations:** Department of Biological Sciences, Kent State University, Kent, OH, United States

**Keywords:** dendritic spine, synapse, ARID1B, KANSL1, WDR5, autism, intellectual disability

## Abstract

Formation of dendritic spine and synapse is an essential final step of brain wiring to establish functional communication in the developing brain. Recent findings have displayed altered dendritic spine and synapse morphogenesis, plasticity, and related molecular mechanisms in animal models and post-mortem human brains of autism spectrum disorders (ASD) and intellectual disability (ID). Many genes and proteins are shown to be associated with spines and synapse development, and therefore neurodevelopmental disorders. In this review, however, particular attention will be given to chromatin modifiers such as AT-Rich Interactive Domain 1B (ARID1B), KAT8 regulatory non-specific lethal (NSL) complex subunit 1 (KANSL1), and WD Repeat Domain 5 (WDR5) which are among strong susceptibility factors for ASD and ID. Emerging evidence highlights the critical status of these chromatin remodeling molecules in dendritic spine morphogenesis and synaptic functions. Molecular and cellular insights of ARID1B, KANSL1, and WDR5 will integrate into our current knowledge in understanding and interpreting the pathogenesis of ASD and ID. Modulation of their activities or levels may be an option for potential therapeutic treatment strategies for these neurodevelopmental conditions.

## Introduction

Autism spectrum disorder (ASD) and intellectual disability (ID) are a wide array of developmental disorders affecting approximately 1.8% and upward of 8% of the population, respectively (Pasca et al., 2021; Moffat et al., 2022). Behavioral phenotypes of ASD often present themselves as trouble socializing and communicating with others, stereotypic behaviors, and niche interests (Guang et al., 2018). Beyond this, ASD has comorbidity with syndromic or non-syndromic ID such as Fragile X Syndrome (FXS) (Guang et al., 2018). ID often affects both intellectual and cognitive functioning of the individual with the disorder and presents an inability or difficulty to take part in day-to-day living (Vasudevan and Suri, 2017). Like ASD, an important note about ID is that there are many variations of intellectual dysfunction: no single disorder falls under this broad term. ASD and ID are mutually influential. For example, FXS is considered one of the most common genetic causes for ASD (Devitt et al., 2015). ASD and ID are diverse developmental disorders in their genetic underpinnings, with a multitude of mutations and haploinsufficiencies of genes directly leading to these neurodevelopmental conditions (Moffat et al., 2019, 2022). A specific way that either developmental disability can occur is through the insufficient amount of specific chromatin modifying factors being present during development (Arbogast et al., 2017).

Abnormal dendrites and dendritic spines dramatically alter brain function (Moffat et al., 2019; Ka et al., 2022). Abnormalities in dendrites and their spines consist of branching pattern, spine density, size, and morphology (Goikolea-Vives and Stolp, 2021). Dendrites and dendritic spines play significant roles for a neuron in forming synapses to receive and process information from other neurons. Synaptic deficits may trigger early events in the pathogenesis of neurodevelopmental disorders (Taoufik et al., 2018; Linda et al., 2022). Many synaptic proteins are part of the pathogenic risk factors of ASD (Jiang et al., 2022). Also, ID target genes such as the *Fragile X Messenger Ribonucleoprotein 1* (*FMR1*) and *Cullin 4B* (*CUL4B*) encode or regulate essential synaptic proteins (Nakagawa and Xiong, 2011; Prieto et al., 2020). The development of dendrites, dendritic spines, and synapses are all directly impacted by chromatin modifying factors during early development (Zollino et al., 2012; Ka et al., 2016; Arbogast et al., 2017; Baronchelli et al., 2017; Moffat et al., 2021). Accordingly, abnormal chromatin modifiers are associated with neurodevelopmental conditions featuring cognitive, social, and other behavioral deficits due to their impact on neuron morphology and function (Moffat et al., 2019, 2022).

Chromatin modifying factors act on DNA in early development to perform several tasks, such as assisting in stem cell specialization as well as neural progenitor formation and migration (Baronchelli et al., 2017). In this review, three specific chromatin modifying factors will be discussed: AT-Rich Interactive Domain 1B (ARID1B), KAT8 regulatory non-specific lethal (NSL) complex subunit 1 (KANSL1), and WD Repeat Domain 5 (WDR5). Evidence has been found that without enough of each of these proteins, there will be developmental abnormalities that will lead to ASD and/or ID as a symptom (Nakagawa and Xiong, 2011; Zollino et al., 2012; Dias et al., 2014; Arbogast et al., 2017; Seabra et al., 2017; Moffat et al., 2019; Carroll et al., 2021; Ka et al., 2022). Recent research has shown that although a deficiency in these proteins leads to similar disorders, the way in which it acts on the molecular level to interfere with neurodevelopment varies greatly (Nakagawa and Xiong, 2011; Zollino et al., 2012; Jung et al., 2017; Ka et al., 2022). This may account for variability within the spectrum of ASD and ID. Through the research and exploration of each of these proteins, evidence of their potential involvement in the development of ASD and ID have been suggested. This will be covered further along with the current animal models of ASD and ID, as well as the pathogenic processes associated with abnormal spines and synapses. Potential pharmacological approaches that target the potential reversal of altered functions of chromatin modifiers will also be discussed.

## Neural connectivity and chromatin remodelers associated with ASD and ID

Brains of people with ASD and/or ID show abnormal morphology of dendritic spines and synapses, compared to a neurotypical brain (Huttenlocher, 1974; Marin-Padilla, 1976; Kaufmann and Moser, 2000; London and Hausser, 2005; Pardo and Eberhart, 2007; Granato and De Giorgio, 2014). This is also a pervasive phenotype across a multitude of ASD and ID mouse models associated with deficient chromatin modifying factors including ARID1B, KANSL1, and WDR5 (Moffat et al., 2019, 2022; Ka et al., 2022). Specifically, the dendrites of pyramidal neurons that lack or contain insufficient chromatin factors are underdeveloped and do not extend to their target area between brain regions as they are expected to Ka et al. (2016) and Ka et al. (2022). Alongside this, the synapses (either inhibitory or excitatory) are morphologically altered significantly and are dysfunctional in their neurotransmission, leading to behavioral deficits recapitulating ASD and ID symptoms. For example, ARID1B haploinsufficiency has less inhibitory dendritic projections and synaptic formation, and in turn diminished inhibitory synaptic transmission (Jung et al., 2017). On the contrary, WDR5 appears to impact excitatory synapses and without it there is an increase in excitatory dendritic branches and synapses (Ka et al., 2022). KANSL1 also appears to impact synapse activity, with evidence of a KANSL1 deficiency leading to an overall decrease in the activity of synapses, as well as impaired synaptic function (Linda et al., 2022). Dendritic spine and synaptic morphologies correlate with the neuronal ability to transmit signals between regions of the brain. This evidence shows that both ARID1B and WDR5 act on dendritic spine and synaptic morphogenesis and/or maintenance, strongly suggesting the deficiency of ARID1B or WDR5 as a causal factor for behavioral deficits of ASD and ID although each deficiency induces an opposing type of cellular mechanism to regulate distinct synapses. MECP2 is another chromatin remodeling protein. A *Mecp2* knockout mouse model has revealed that synapses involved in memory between the ventral hippocampus and medial prefrontal cortex are impaired in ASD (Phillips et al., 2019). Although MECP2 is not the primary focus of this review, it is important to note that MECP2 is genetically linked to neurodevelopmental disorders with a spectrum of phenotypes that fall under the umbrella of ASD and ID (Nakagawa and Xiong, 2011).

Interestingly, as noted in a previous study involving WDR5 (Ka et al., 2022), the abnormal spine or synapse number doesn’t encapsulate a decrease, but instead an increase. However, the increase appears to have a negative impact on synapse formation and function. For example, although there is an overabundance of dendrites and spines in the *Wdr5* knockdown model, the dendritic spines are underdeveloped (Ka et al., 2022). These underdeveloped synapses are unlikely to receive proper synaptic inputs. Furthermore, the increase in spines and synapses is limited to a certain type of synapse that in this case is an excitatory synapse. These specific deficits may disrupt excitatory neurotransmission and lead to excitation/inhibition imbalance often shown in ID and/or ASD.

The chromatin remodeling factors, ARID1B, KANSL1, and WDR5, are also associated with a multitude of syndromic neurodevelopmental conditions that display ID features. These include Coffin-Siris syndrome (ARID1B), Koolen-de Vries syndrome (KANSL1), Kabuki syndrome (WDR5), and Kleefstra syndrome (WDR5) (Nakagawa and Xiong, 2011; Koolen et al., 2016; Ka et al., 2022; Moffat et al., 2022). Each of these syndromes is associated with abnormal dendrite, dendritic spine, and/or synaptic development (Ka et al., 2016, 2022; Linda et al., 2022). This review will further delve into the role of these chromatin remodelers on spine/synaptic morphology and associated molecular mechanisms as well as their behavioral impacts by discussing recent studies using genetic animal models.

## Role of ARID1B in neurological behaviors, dendritic spine and synapse development, and associated molecular signaling

**FIGURE 1:**
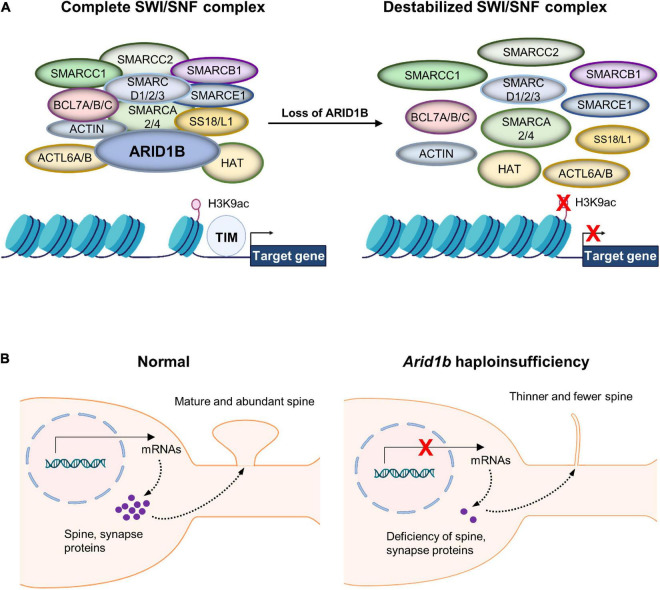
Chromatin remodeling by the BRG1/BRM-associated factor (BAF) complex. **(A)** AT-Rich Interactive Domain 1B (ARID1B) acts as a scaffolding protein that holds together the BAF complex, giving it the ability to interact with chromatin. Without ARID1B, the complex is destabilized and therefore unlikely to remodel chromatin correctly. The BAF complex regulates nuclear gene transcription by remodeling a chromatin structure that contains its target genes. The complex performs histone modifications including acetylation of histone H3 at lysine 9 (H3K9ac). Thereby, chromatin detangles and exposes naked gene areas to the transcription initiation machinery (TIM). Multiple enzymes are collectively involved in the histone modification at the regulatory regions of their target genes. Although histone H3 trimethylation at lysine 4 (H3K4me3) is also impacted by the BAF complex as well as histone H3 trimethylation at lysine 27 (H3K27me3), they are not featured in this specific example. **(B)** Normal neurons express spine and synaptic proteins that move to synaptic compartments and direct synaptic maturation. However, *Arid1b* haploinsufficient neurons lack proper expression of synapse-associated genes, leading to underdeveloped dendritic spines.

AT-Rich Interactive Domain 1B is a DNA-binding subunit of the BRG1/BRM-associated factor (BAF) chromatin remodeling complex and serves as a scaffolding protein to hold the BAF complex components together (Figure 1A; Okawa et al., 2017; Seabra et al., 2017; Sato et al., 2018). Subunits of the BAF complex include: BCL7A/B/C, BAF Chromatin Remodeling Complex; SMARC A/B/C/D/E 1/2/3, SWI/SNF Related Matrix Associated Actin Dependent Regulator of Chromatin Subfamily (A/B/C/D/E) Member (1/2/3/4); ACTL6A/B, Actin Like 6 (A/B); SS18L1, Synovial Sarcoma Translocation Gene on Chromosome 18-Like 1 (Figure 1A). ARID1B contributes to gene expression by regulating transcription and multiple downstream cellular processes (Seabra et al., 2017). While ARID1B is involved in a myriad of developmental events including cell proliferation and differentiation, it particularly plays an important role in dendritic arborization and synapse formation (Nagl et al., 2005, 2007; Ka et al., 2016; Jung et al., 2017; Moffat et al., 2019, 2022). Accordingly, *ARID1B* gene alterations have been associated with neurodevelopmental defects. ARID1B deficiency in the brain leads to ASD, ID, and some syndromic developmental disorders such as Coffin-Siris syndrome (Vasudevan and Suri, 2017; Guang et al., 2018; Moffat et al., 2019, 2022). Most mutations found in the *ARID1B* gene create *ARID1B* haploinsufficiency (Halgren et al., 2012; Hoyer et al., 2012; Santen et al., 2012). Either a non-sense point mutation or an insertion/deletion mutation can generate a frame shift of the *ARID1B* gene, leading to an early truncation of the full ARID1B protein. This half-copy gene alteration defines *ARID1B* haploinsufficiency and is induced by both inheritable and *de novo* gene variations (Deciphering Developmental Disorders Study, 2015; Wieczorek, 2018; Rylaarsdam and Guemez-Gamboa, 2019).

To investigate pathological mechanisms underlying *ARID1B* haploinsufficiency-associated ASD and ID, four research groups have generated mouse models. Celen et al. (2017), Shibutani et al. (2017), and Ellegood et al. (2021) used the CRISPR/Cas9 gene editing system to establish *Arid1b* haploinsufficient (heterozygous) mutant mice, while Jung et al. (2017) employed a knockout first allele strategy. While Shibutani et al. (2017) deleted exon 3 of the *Arid1b* gene, other three groups removed exon 5. Each group implemented a variety of assays to characterize the behavioral phenotypes of *Arid1b* haploinsufficient mice (Table 1; Celen et al., 2017; Jung et al., 2017; Shibutani et al., 2017; Ellegood et al., 2021). To assess changes in social communication, Celen et al. (2017) and Ellegood et al. (2021) examined ultrasonic vocalizations (USVs). They found that *Arid1b* haploinsufficient mice emit altered USVs compared to wild type controls. Further expanding upon social communication, Jung et al. (2017) used the three-chamber and open field tests to show a decrease in social exploration and social memory in *Arid1b* haploinsufficient mice. In addition, Celen et al. (2017) and Shibutani et al. (2017) reported that the mutant mice show decreased reciprocal social interaction in a home cage. Ellegood et al. (2021) also revealed a decrease in social dyad interaction between male and female in an open field. When testing repetitive behavior that is one of the core ASD symptoms, Celen et al. (2017) and Jung et al. (2017) reported an increase in time spent self-grooming in *Arid1b* haploinsufficient mice, while other groups showed no change in the behavior (Shibutani et al., 2017; Ellegood et al., 2021).

**TABLE 1:** Comparison of behavioral phenotypes observed in AT-Rich Interactive Domain 1B *(Arid1b)* mouse lines.

Behavioral test	Celen et al., 2017	Jung et al., 2017	Shibutani et al., 2017	Ellegood et al., 2021
**Social**				
● Three-chamber (social exploration)	N/A	Decreased	No change	No change
● Three-chamber (social novelty)	N/A	Decreased	No change	No change
● Home cage social (reciprocal sociability)	Decreased	N/A	Decreased	N/A
● Open field social (reciprocal sociability)	N/A	Decreased	No change	N/A
● Open field social (male-female social dyad interaction)	N/A	N/A	N/A	Decreased
● Ultrasonic vocalizations (social communication in pups)	Impaired	N/A	N/A	Impaired
**Repetitive**				
● Grooming	Increased	Increased	No change	No change
**Learning and memory**				
● Novel object recognition (recognition memory)	N/A	Impaired	N/A	Impaired
● Morris water maze (spatial learning and memory)	No change	Impaired	N/A	N/A
● Barnes maze (spatial learning and memory)	N/A	N/A	No change	N/A
● T-Maze (spatial memory)	N/A	Impaired	No change	N/A
● Fear conditioning (fear-related learning and memory)	No change	N/A	Increased	No change
● Rotarod test (motor learning)	N/A	Impaired	N/A	N/A
**Anxiety-like**				
● Elevated plus maze	Increased	Increased	Increased	Increased
● Open field	Increased	Increased	N/A	N/A
● Light-dark Box	Increased	N/A	No change	No change
**Depression-like**				
● Forced swim	N/A	Increased	N/A	N/A
● Tail suspension	N/A	Increased	N/A	N/A

Regarding learning and memory behavior, Jung et al. (2017) and Ellegood et al. (2021) reported that *Arid1b* heterozygous mice show impaired spatial, recognition, and motor learning and retention memory. Shibutani et al. (2017) also confirmed learning and memory dysfunction that *Arid1b* heterozygous mice display enhanced performance in a fear conditioning test. But the defective fear learning in *Arid1b* mutant mice was not observed by the groups of Celen et al. (2017) and Ellegood et al. (2021). Different animal handling and experimental environments among laboratories may lead to the observed discrepancies. Together, these behavioral experiments across the several independent groups indicate that the insufficient amount of ARID1B leads to social communication and learning deficits in mice as seen in humans.

Altered emotion is a well-known comorbid factor in people with ASD and/or ID. The four groups used the elevated plus maze test to assess anxiety-like behavior. The consensus is that *Arid1b* haploinsufficient mice spend less time in and show a lower number of total entries into the open arms in the elevated plus maze test (Celen et al., 2017; Jung et al., 2017; Shibutani et al., 2017; Ellegood et al., 2021). In another anxiety assessment using the open field test, *Arid1b* haploinsufficient mice spend less time in, and have fewer entries into the center of the arena (Celen et al., 2017; Jung et al., 2017). In addition, Celen et al. (2017) reported that mutant mice explore the brightly lit section less in the light-dark box test, while Shibutani et al. (2017) and Ellegood et al. (2021) identified no changes in exploratory behavior compared to control mice. In assessing depression-like behavior, Jung et al. (2017) reported that *Arid1b* haploinsufficient mice exhibit increased immobilization behavior in the forced swim and tail suspension test (Ellegood et al., 2021).

There is a strong correlation between dendritic spine morphology and synapse formation and function. Dendritic spines contribute to synaptic transmission (Matsuzaki et al., 2001), synapse formation, spine stability (Holtmaat et al., 2005), and Ca2^+^ diffusion from the spine head to the parent dendrite (Matsuzaki et al., 2001; Korkotian et al., 2004; Holtmaat et al., 2005; Noguchi et al., 2005). *Arid1b* knockdown in mice suppresses dendrite arborization in pyramidal neurons specifically in the cortex and hippocampus (Ka et al., 2016), suggesting ARID1B’s key status in the establishment of neuronal wiring and communication. The dendritic defect may cause an inability for them to properly receive signals from presynaptic neurons. Using *in utero* electroporation of *Arid1b* shRNA into the developing mouse brain, Ka et al. (2016) suppressed *Arid1b* expression in the ventricular region of the cerebral cortex where newly generated pyramidal neurons commence radial migration. They found a marked disruption of apical dendrites in *Arid1b*-deficient neurons. The numbers and lengths of primary and secondary apical dendrites in *Arid1b*-deficient neurons are reduced in the developing brain compared to normal cells (Ka et al., 2016). This abnormal dendrite morphology continues during post-natal periods. Ka et al. (2016) also observed abnormal dendritic orientation in *Arid1b*-deficient neurons. While normal neurons extend basal dendrites laterally and toward the ventricular zone, *Arid1b*-deficient neurons orient their basal dendrites more laterally. This is a notable phenotype because the orientation of dendritic branches contributes to the establishment of local connectivity (Ka et al., 2016). It is noted that the abnormal development of dendrites accompanies a decrease in dendritic outgrowth into cortical layer I (Ka et al., 2016). Dendritic innervation into layer I is important in regards to neural input receptions from local inhibitory interneurons as well as long-range axons of subcortical neurons. Thus, abnormal dendritic outgrowth and orientation may pose impaired development of neural circuits associated with *Arid1b* deficiency-induced cognitive and social deficits. Furthermore, *Arid1b*-deficient neurons show abnormal spines (Ka et al., 2016). The number and length of *Arid1b*-deficient dendritic spines are reduced compared to normal spines. The width of spine heads is decreased as well, suggesting their immature status.

Synapses are the functional connections that allow electrical or chemical signals to flow from the pre-synaptic neuron to the post-synaptic neuron. Disruption in synaptic development has been considered as a potential mechanism for ASD and/or ID pathogenesis (Mellgren and Coulson, 1983). As expected by the altered dendrites and spines, *Arid1b* haploinsufficiency displays abnormal synaptic formation and transmission in the brain (Jung et al., 2017). For example, *Arid1b* haploinsufficient mice show an expanded synaptic cleft as well as a shortened post-synaptic length (Jung et al., 2017; Moffat et al., 2022). It is important to note, however, these synaptic deficits appear to be specific to inhibitory neurons as they are not seen in excitatory neurons (Jung et al., 2017; Carroll et al., 2021). The number of inhibitory synapses is decreased while the excitatory synapse number is normal in *Arid1b*-deficient brains (Jung et al., 2017). Synaptic transmission measured by miniature post-synaptic currents confirms the inhibitory-specific defects. The aberrant dendrite and synapse appear to be related to ARID1B’s natural role in gene expression regulation as *Arid1b* deficiency leads to aberrant expression of synapse-associated factors including c-Fos and Arc (Ka et al., 2016). Chromatin immunoprecipitation experiments with an ARID1B antibody shows that ARID1B physically binds to the promoters of c-Fos and Arc genes (Ka et al., 2016), suggesting the potential role of gene expression alteration in mediating *Arid1b* deficiency-induced dendrite and synapse dysfunction (Figure 1B). Thus, restoring the level of ARID1B targets may be functionally relevant. Indeed, overexpression of c-Fos or Arc in *Arid1b*-deficient neurons rescues dendritic abnormality (Ka et al., 2016).

By regulating chromatin remodeling and transcription factor access to gene regulatory regions, the ARID1B-containing BAF complex orchestrates gene expression for a multitude of neurodevelopmental processes, including neural stem cell proliferation, neuronal migration, and circuit formation (Jung et al., 2017; Moffat et al., 2022). As such, the impact of *Arid1b* haploinsufficiency on dendrite and synapse development is thought to be linked to the ARID1B’s role in gene expression regulation. These neurodevelopmental processes require gene expression to provide raw materials for spine and synapse construction and further maintenance and dynamics. Indeed, an ARID1B deficiency has been demonstrated to alter a multitude of genes whose expression promotes neurite outgrowth, including *Gap43*, *Gprn1*, and *Stmn2* genes (Ka et al., 2016). Many critical questions need to be addressed in future studies. For example, it largely remains unknown what genes are primarily targeted by ARID1B, how ARID1B specifically selects genes to turn on and off, and what cellular and molecular mechanisms mediate ARID1B functions. These previous studies represent early but significant progress made in mouse models toward the molecular interplay between ARID1B and histone molecules. Within ARID1B deficient neurons, there is an incapability for transcription regulation to take place (Jung et al., 2017). This is due to decreased levels of histone H3 acetylation at lysine 9 (H3K9ac) and histone H3 trimethylation at lysine 4 (H3K4me3), which both are transcription activators, in the *Arid1b* haploinsufficient brain compared to the control. On the contrary, transcriptional repressor histone H3 trimethylation at lysine 27 (H3K27me3) showed a significant increase (Jung et al., 2017). This shows a clear connection between ARID1B deficiency and changes in epigenetic modifications. As suggested by its impact on inhibitory neurons such as parvalbumin interneurons, *Arid1b* haploinsufficiency decreases the expression of the *parvalbumin* (*Pvalb*) gene (Jung et al., 2017). More importantly, *Arid1b* haploinsufficiency suppresses the epigenetic modification of H3K9ac within the *Pvalb* promoter region (Figure 1A). This disruption of histone modification appears to create an unfavorable environment for gene expression because the transcription initiation step at the *Pvalb* promoter is blocked by *Arid1b* haploinsufficiency (Jung et al., 2017). This prior research is certainly exciting, but there are more questions remaining. Nothing is known about whether this type of abnormal transcription takes place at other gene loci directly involved in spine or synapse formation and function. The fact that *Arid1b* deficiency alters the expression of Wnt-β-catenin signaling components (Jung et al., 2017) may shed light on a potential role of ARID1B in synapse gene expression.

## Role of KANSL1 in neurological behavior, dendritic spine and synapse development, and associated molecular signaling

**FIGURE 2:**
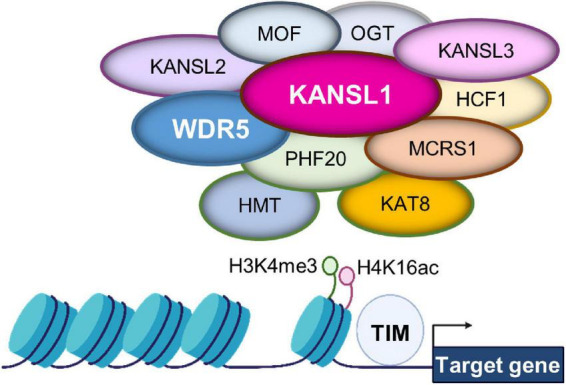
Gene expression regulation by the non-specific lethal (NSL) complex at chromatin. The NSL complex regulates nuclear gene transcription by modifying histone H4 at lysine 16 (H4K16) and histone H3 at lysine 4 (H3K4). The complex contains multiple histone modifying enzymes and co-factors, including histone methyltransferase (HMT) and KAT8. Thus, the access of transcription factors is regulated for appropriate gene expression. These histone modifications are supported by KAT8 regulatory non-specific lethal (NSL) complex subunit 1 (KANSL1) and/or WD Repeat Domain 5 (WDR5).

Chromatin structure undergoes dynamic changes by a combination of post-translational modifications on DNA and histones. Among the chromatin modification, histone 4 lysine 16 acetylation (H4K16ac) is a particularly notable chromatin structure (Figure 2; Sheikh and Akhtar, 2019). The level of lysine acetylation is controlled by lysine acetyltransferases (KATs). Acetylation can have a range of effects on target proteins, including changes in cellular location (Li et al., 2020). KATs can also regulate post-translational phosphorylation modifications (Li et al., 2020). Several deacetylases such as histone deacetylase 1 (HDAC1) and HDAC2 act to counter the KAT activity by deacetylating K16 in the H4 molecule in the developing brain (Li et al., 2019). KANSL1 is a scaffolding protein within the NSL complex that contains KATs (Dias et al., 2014; Sheikh and Akhtar, 2019; Sheikh et al., 2019). Other factors associated with the complex include: MOF, Lysine Acetyltransferase 8 (KAT8); OGT, O-linked N-Acetylglucosamine Transferase; HCFC1, Host Cell Factor C1; MCRS1, Microspherule Protein 1; PHF20, PHD Finger Protein 20 (Figure 2). *KANSL1* haploinsufficiency is associated with the neurodevelopmental disorder Koolen-de Vries syndrome (KdVS), also known as 17q21.31 microdeletion syndrome (Zollino et al., 2012). This is a rare monogenic disorder characterized by moderate ID, developmental delay, epilepsy, and friendly behavior (Li et al., 2022). Recent studies using a *Kansl1* heterozygous knockout mouse showed that *Kansl1* haploinsufficiency results in a significant impairment of recognition memory compared to the normal wild type condition in the object recognition test (Arbogast et al., 2017; Li et al., 2022). Additionally, *Kansl1* haploinsufficiency disrupts spatial memory as shown in the increases in the escape time and the number of platform paths during the Morris water maze test (Arbogast et al., 2017; Li et al., 2022). *Kansl1* haploinsufficiency specifically induces these learning and memory deficits because it has no impact on motor function as both wild type and *Kansl1* mutant mice show no significant difference in the swimming distance (Arbogast et al., 2017; Li et al., 2022). These animal studies strongly suggest that *KANSL1* gene mutations are a clear manifestation of the KdVS’s ID phenotype.

In the KdVS mouse model, KANSL1 deficiency has been shown to result in impaired synaptic transmission in the hippocampus (Arbogast et al., 2017). The impaired synaptic function is associated with a decrease in the number of spines in the CA1 region of the hippocampus. This hippocampal dysfunction particularly at the synaptic level is consistent with the memory deficit observed in the *Kansl1* haploinsufficient mouse model (Arbogast et al., 2017; Li et al., 2022). Mechanistically, autophagy malfunction is implicated in the synaptic dysfunction caused by *Kansl1* haploinsufficiency (Li et al., 2022; Linda et al., 2022). Cellular aggregation of misfolded proteins is the most common pathological hallmark of many neurodevelopmental and neurodegenerative diseases. Autophagy is one of the major intracellular machineries to eliminate misfolded proteins (Linda et al., 2022). It plays a vital role in a variety of biological events including development, apoptosis, pathogen infection, immune response, and degeneration. In neurons, autophagy contributes to synaptic plasticity and memory formation (Guo et al., 2018). Autophagy protects against the progression of neurodegeneration, potentially saving the structure and function of synapses (Linda et al., 2022). Problems in autophagic events often accumulate reactive oxygen species (ROS), which adversely influences synaptic function. *Kansl1* haploinsufficient mice demonstrate autophagy dysfunction and in turn show ROS accumulation and mitochondrial malfunction (Li et al., 2022). Consistently, deficits in autophagy, ROS regulation, and synaptic dysfunction are observed in human induced-pluripotent stem cells (hiPSCs) derived from KdVS patients (Li et al., 2022; Linda et al., 2022). The *KANSL1* hiPSCs show significantly lower lysosomal activity inside of the cells (Li et al., 2022). This leads to an imbalance in autophagy regulation within the KdVS cells (Li et al., 2020; Linda et al., 2022). Thus, KANSL1 appears to be required for normal synaptic plasticity through preserving autophagy to induce synapse-specific breakdown of proteins. With insufficient KANSL1, neurons lack balanced autophagy, increase ROS and mitochondrial damages, leading to synapse dysfunction (Li et al., 2022; Linda et al., 2022).

It is noted that *Kansl1* haploinsufficient neurons display an increase in mTOR phosphorylation and LC3 accumulation (Linda et al., 2022). So, the mTOR molecular pathway is thought to be involved in *Kansl1* haploinsufficiency. Indeed, the treatment with rapamycin–which is an inhibitor of mTOR–significantly reduces the level of H4K16ac in control neurons but has no effect in *Kansl1*–haploinsufficient cells (Linda et al., 2022). This suggests that mTOR mediates the KANSL1–induced epigenetic modification of histone acetylation (Li et al., 2022; Linda et al., 2022). Still, it is unclear whether the mTOR pathway is involved in the regulation of KANSL1–dependent epigenetic modification of histones within synapse genes. This remains an important question to be answered in future studies. If so, it will expand our understanding toward the *KANSL1* haploinsufficiency mechanism of synaptic dysregulation and memory deficits.

## Role of WDR5 in dendritic spine and synapse development

WD Repeat Domain 5, a member of the WD-40 group of proteins, is considered a highly conserved molecule and involved in multiple pathologies ranging from cancer to neurodevelopmental disorders as well as neurodegenerative diseases such as Huntington’s disease (Baronchelli et al., 2017; Guarnaccia and Tansey, 2018). Dysfunctional WDR5 due to genetic mutations is particularly linked to Kabuki syndrome and Kleefstra syndrome characterized by ID (Lavery et al., 2020; Ka et al., 2022). WDR5 has an ability to multitask within organisms. Most significant to the current literature, WDR5 participates in chromatin modifying complexes as an adaptor protein (Guarnaccia and Tansey, 2018). For example, WDR5 is a partner protein in the NSL complex that contains KANSL1 and KATs (Dias et al., 2014; Sheikh and Akhtar, 2019; Sheikh et al., 2019). Thus, WDR5 appears to collaborate with KANSL1 to help the KAT function in H4 lysine acetylation (Figure 2). Within a chromatin modifying complex, WDR5 can bind with over 2 dozen direct interaction partners, and all these partners bind to one of two sites: the WDR5 binding motif or the WDR5-interacting site (Guarnaccia and Tansey, 2018). The multitude of interaction appears to allow WDR5 to operate within a diverse range of biological contexts (Baronchelli et al., 2017; Guarnaccia and Tansey, 2018; Ka et al., 2022).

One of the well-known chromatin modifying complexes that contain WDR5 is the histone methyltransferase (HMT) complex that directs H3K4 dimethylation (H3K4me2) and trimethylation (H3K4me3) (Guarnaccia and Tansey, 2018; Ka et al., 2022). It has been shown that suppression of WDR5 blocks H3K4 trimethylation and differentiation of embryonic stem cells (Wang et al., 2011). This study suggests a role of WDR5 in differentiation of other cell types such as neurons. Consistent with this idea, recent studies have shown that WDR5 regulates neuronal differentiation (Baronchelli et al., 2017; Ka et al., 2022; Linda et al., 2022). Baronchelli et al. (2017) showed a WDR5 role in the differentiation of human induced pluripotent stem cells derived from a patient with Huntington’s disease (HD-hiPSCs). In the hiPSCs, WDR5 is hypermethylated, which leads to down-modulation of encoded protein expression. This would decrease stem cell differentiation and specialization into neurons, causing a disruption in neuronal development in human cell model. There is more detailed evidence for the WDR5’s role in neuronal differentiation in mice. For example, WDR5-deficiency suppresses the multipolar-to-bipolar transition of migrating neurons in the developing mouse brain (Ka et al., 2022). In a similar vein, WDR5-deficient neurons show abnormal dendritic polarity in cortical pyramidal neurons compared to normal neurons (Ka et al., 2022). Instead of having a typically long apical dendrite, WDR5-deficient neurons display several short apical dendrites. These primary apical dendrites do not extend into the pia where they receive and integrate neuronal information (Ka et al., 2022). While the length of primary apical dendrites is found to be decreased in WDR5-deficient neurons, the length of primary basal dendrites is increased. Furthermore, WDR5-deficient neurons show an increase in the number of dendritic spines compared to normal neurons in the developing mouse brain (Ka et al., 2022). However, the abundance of dendritic spines in WDR5-deficient neurons do not appear to coincide with functional maturation as the spines have smaller heads and shorter necks (Ka et al., 2022). WDR5-deficient neurons also show abnormal synapses as the number of synapses is increased compared to normal neurons (Ka et al., 2022). However, this synaptic alteration is quite specific to excitatory synapses and not shared with inhibitory synapses. This may represent a broken balance between excitation and inhibition in the WDR5-deficient brain and create an irregular neurotransmission. Combining with the abnormal dendritic polarity, the altered spine development in WDR5-deficient neurons seems to generate improper communication between brain regions. This may be a pathological mechanism underlying ID phenotypes in Kabuki syndrome and Kleefstra syndrome.

Still, research into WDR5 and its involvement in ASD and ID is in its infancy. There is no behavioral mouse model available for characterizing phenotypes of *WDR5* human mutations of any form. This may be an instrumental next step in responding to WDR5-associated neurodevelopmental disorders if future research goes in similar directions as ARID1B and KANSL1 (Arbogast et al., 2017; Moffat et al., 2021).

## Reversing dendritic spine and synapse abnormality as a potential therapeutic option for chromatin modifier-associated neurodevelopmental conditions

Neurodevelopmental disorders are a complex disease caused by a combination of different etiological factors that include abnormal molecular signaling pathways, synapses, or brain connections (Jiang et al., 2022). Clinical trials of pharmacological interventions for neurodevelopmental diseases are challenging and at an early stage, with questions to be resolved about outcome measures, age of treatment onset, and duration of treatment (Jacquemont et al., 2018; Rhine et al., 2019; Basilicata et al., 2021). While many biological factors are of therapeutic targets, a reversal of dendrite or spine abnormality could offer an option for chromatin modifier-associated ASD and/or ID. Since all ARID1B, KANSL1, and WDR5 pathologies are a haploinsufficiency issue, replenishing the level of these factors may rescue morphological and functional phenotypes. In a pre-clinical study, WDR5 overexpression in WDR5-deficient neurons restores the dendritic outgrowth and polarity in the mouse brain (Ka et al., 2022). The timing and location of overexpression certainly adds a degree of challenge in humans, but virus-mediated technology may be utilized as in other non-neurological diseases.

Synapse function may also be targeted for chromatin modifier-associated neurological conditions. Using an *Arid1b* haploinsufficient mouse model, Jung et al. (2017) demonstrated that treatment with a low dose of the GABA_A_ receptor positive allosteric modulator, clonazepam, is effective in reversing inhibitory synaptic transmission as well as several ASD-like behaviors. This approach is based on the idea that neurodevelopmental disorders, particularly ASD, are caused by the disrupted homeostatic mechanism that leads to synaptic imbalance between excitation and inhibition (Han et al., 2014; Jung et al., 2017; Goikolea-Vives and Stolp, 2021; Moffat et al., 2021, 2022). The insufficient tone of inhibitory synapse is an easy target point for pharmacological manipulation as there are drugs available in clinics.

Histone modifications such as acetylation or methylation are another potential therapeutic target for chromatin modifier-associated ASD or ID. Mouse models described in this review show altered histone modifications in the brain (Jung et al., 2017; Ka et al., 2022). Small molecular inhibitors of numerous histone acetylases or histone deacetylases are already in clinical use for cancers. Yet, there is no pre-clinical or clinical evidence to demonstrate this possibility for ASD and/or ID.

A recent study has also found evidence that the use of fluoxetine–a selective serotonin reuptake inhibitor (SSRI)–in early life appears to impact behavior specifically in the *Arid1b* mouse model (Kim et al., 2022). It appears that fluoxetine impacts transcription factors on the molecular level, which then alter synaptic morphology. Thus, *Arid1b* mice do not respond to the same degree that they typically would without fluoxetine (Kim et al., 2022). The utilization of an SSRI chronically from days 3 to 21 in an ASD mouse model has shown promise as a way of addressing behavioral phenotypes associated with autism-like behavior in mice (Kim et al., 2022).

Lastly, neurotrophic factors such as BDNF may be considered to enhance synaptic transmission for chromatin modifier-associated ASD since they are linked to ASD and have a critical role in synapse formation and function (Chapleau et al., 2009). But they pose a risk in penetrating the blood brain barrier. Also, it is unclear how they selectively target either excitatory or inhibitory synapse. Similarly, low doses of ketamine increase the number of spines and synapses (Li et al., 2020), but again the selectivity is still an issue for this drug.

## Conclusion

Haploinsufficiency of chromatin remodelers such as ADRI1B, KANSL1, or WDR5 is linked to ASD and ID. The roles of these factors in neuronal connectivity and communication have been identified in mouse models, although further studies with more animal models and humans are required to solidify those results. They regulate dendrite and synapse development that contribute to the formation of the healthy brain. Thereby, therapeutic intervention through spine and synapse morphology and function could be explored to possibly assist in treating behavioral phenotypes of chromatin modifier-associated ASD and ID. Ongoing and future research must consider the molecular mechanism regarding how they impact behavioral phenotypes by zooming out to the big picture, beginning with chromatin being altered by this array of proteins.

## Author contributions

All authors listed have made a substantial, direct, and intellectual contribution to the work, and approved it for publication.
